# *Large* is required for normal astrocyte migration and retinal vasculature development

**DOI:** 10.1186/s13578-017-0143-9

**Published:** 2017-04-17

**Authors:** Min Zhou, Herui Wang, Hui Ren, Rui Jiang, Chi Zhang, Xiaohui Wu, Gezhi Xu

**Affiliations:** 1 Department of Ophthalmology, Eye and ENT Hospital of Fudan University, Shanghai, 200031 China; 2 Shanghai the Key Laboratory of Visual Impairment and Restoration, Fudan University, Shanghai, China; 3 State Key Laboratory of Genetic Engineering and National Center for International Research of Development and Disease, Institute of Developmental Biology and Molecular Medicine, Innovation Center of Genetics and Development, School of Life Sciences, Fudan University, Shanghai, 200433 China; 4 Neuro-Oncology Branch, National Cancer Institute, National Institutes of Health, Bethesda, MD USA

**Keywords:** Persistent fetal vasculature (PFV), *Large*, Inner limiting membrane (ILM), Retinal astrocytes

## Abstract

**Background:**

Persistent fetal vasculature (PFV) is a congenital developmental anomaly of the eye that accounts for about 5% of childhood blindness. The molecular mechanism of PFV remains unclear. As a glycosyltransferase of α-dystroglycan, *LARGE* mutations have been found in congenital muscular dystrophy patients with brain abnormalities. Spontaneous *Large* mutant mice displayed similar symptoms of human muscle–eye–brain disorders. However, the detailed roles of *Large* in ocular vasculature development still need to be uncovered.

**Results:**

In this paper, we report that a novel *Large* mutation generated by the *piggyBac* transposon insertion leads to PFV and abnormal retinal vasculature in mice. Glycosylation of α-DG, an essential component of the extracellular matrix, was significantly impaired in these *Large* mutants, leading to broken inner limiting membrane (ILM). As a guide of the retinal vasculature development, the distribution of retinal astrocytes became irregular within the retina, and many astrocytes abnormally migrated into the vitreous along with the hyaloid vessels in *Large* mutants.

**Conclusions:**

*Large* is essential for ILM formation and retinal astrocyte migration. The novel *Large* mutant mouse can serve as a new PFV model to further dissect LARGE functions in ocular vasculature development.

## Background

During early fetal development, hyaloid vasculature arises from the optic nerve head, extends through the vitreous, and surrounds the developing lens [1]. Later the fetal vasculature normally regresses and is replaced by retinal vasculature (around mid-gestation in humans and around birth in rodents), resulting in an optically clear path between the cornea and the retina [1]. Failure of the hyaloid vascular regression could lead to persistent fetal vasculature (PFV), a congenital developmental disorder of the eye that accounts for approximately 5% of the childhood blindness. Until now, the mechanisms underlying PFV formation remain unclear.

The inner limiting membrane (ILM) is a basement membrane that defines the border between the retina and the vitreous cavity. The presence and integrity of ILM is essential for normal astrocyte migration and retinal vasculature development [2]. Retinal astrocytes forms a cellular network that provides a template for endothelial cell migration during angiogenesis [1]. *M*utation of *Lama1*, which encodes a basement membrane protein LAMININ α1, disrupts retinal vasculature development and inner limiting membrane formation, leading to vitreoretinal blood vessel formation, persistence of fetal vasculature, and epiretinal membrane formation in mice [3, 4]. These results indicate the pivotal roles of LAMININ in ILM formation and retinal vasculature development.

The interaction between α-dystroglycan (α-DG, a laminin receptor) and laminin is indispensable for the assembly and maintenance of ILM [5]. Correct glycosylation of α-DG is essential for the interaction. Similar with *Lama1* mutants, mutations in α-DG and in an enzyme that participates in glycosylation of α-DG (POMGnT1) also displayed defective ILM formation, abnormal astrocyte distribution and blood vessel formation [6, 7].

Except for POMGnT1, LARGE is another reported glycosyltransferase of α-DG [8]. *LARGE* mutations have been found in congenital muscular dystrophy patients with brain abnormalities [9]. *Myd* mice that carry a spontaneous deletion in *Large* (*Large* ^*myd*^), showed skeletal, cardiac, and tongue muscle dystrophies, defective retinal transmission, and neuronal migration defects, mimicking the human muscle–eye–brain disorders [10, 11]. Another intragenic deletion allele of *Large* (*Large* ^*myd*^) showed ocular vascular defects, including vitreal fibroplasia and retinal vessel tortuosity and fluorescein leakage [7]. However, it’s still unclear about the details how *Large* mutation causes defective ocular vasculature.

Human genetics showed that the severity of the affected patients depends on the LARGE gene mutation type. A patient of Walker–Warburg syndrome, a severe form of dystroglycanopathy was reported to carry a homozygous intragenic loss-of-function deletion in *LARGE* [12]. A less severely affected patient carried a compound heterozygous missense mutation and a heterozygous 1 bp insertion in *LARGE* [9]. The residual function of mutant LARGE protein may be the reason for the milder phenotype in the second patient. Despite of the previously reported *Large* mutant mouse models, new *Large* mutants with different mutation types can expand our understanding of its role in the disease development.

In this study, we report a novel *Large* mutant (*Large* ^*PB*^) mouse line that shows defective retinal vasculature and persistent hyaloid vessels. Hypo-glycosylation of α-DG was found in mutant retina, leading to broken ILM. Retinal astrocytes abnormally migrate through ILM into vitreous along with the persistent hyaloid vessels in the mutants. These features make this mutant a useful model for further dissecting the roles of LARGE in ocular vasculature development.

## Results

### Persistent fetal vasculature (PFV) in *Large* mutant mice

**Fig. 1:**
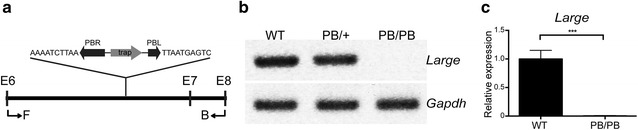
*PB* insertion disrupted *Large* expression. **a** Schematic representation of partial *Large* genomic sequence from exon 6 (E6) to exon 8 (E8) and the insertion site of *PB* transposon. The genomic sequences adjacent to PB repeat termini are labeled next to *PB* transposon. Reverse transcription PCR (RT-PCR) primers are also labeled as F and B. PBR, PB repeat right termini. PBL, PB repeat left termini. Trap, gene trap element. **b** RT-PCR showed disrupted *Large* expression in the retinas of 2-month old *Large* ^*PB/PB*^ mice. **c** Quantitative real-time RT-PCR confirmed complete disruption of normal *Large* transcription in the retinas of 2-month old *Large* ^*PB/PB*^ mice

To investigate the physiological roles of *Large* in retinal vasculature development and hyaloid vessel regression, we analyzed a *Large* mutant (*Large* ^*PB*^) generated in a large-scale mutagenesis project with the *piggyBac* transposon (*PB*) [13, 14]. In this mutant, a *PB* insertion in the sixth intron efficiently disrupted *Large* expression in the retinas of 2-month old homozygous (*Large* ^*PB/PB*^) mice (Fig. 1a–c). Unlike previous reported *Large* ^*myd*^ mice, *Large* ^*PB/PB*^ mice did not show shuffling gait or abnormal posturing when suspended by the tail, indicating milder muscular defects in *Large* ^*PB/PB*^ mice.

**Fig. 2:**
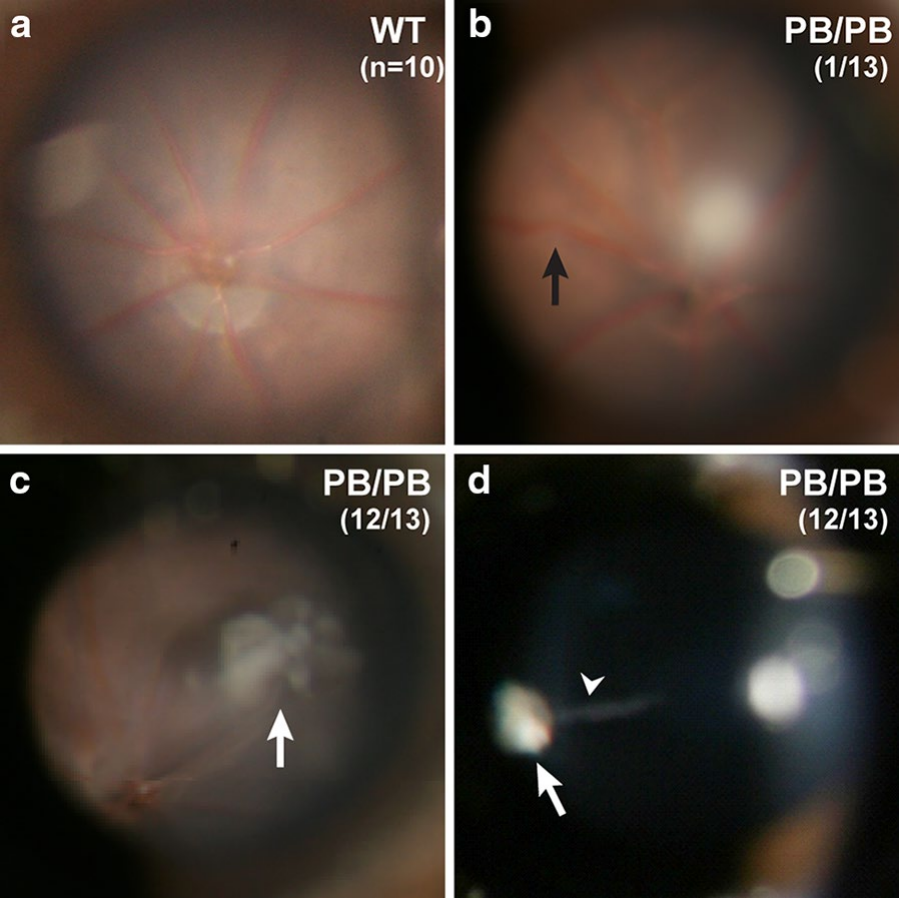
Vitreal and retinal vasculature abnormalities in *Large* ^*PB/PB*^ mutants. **a** All of ten 2-month old wild-type mice have normal fundus. **b**–**d** One out of thirteen 2-month old *Large* ^*PB/PB*^ mutant mice showed retinal vessel tortuosity (**b**, *black arrow*), and the other twelve mutant mice exhibited vitreal fibroplasia (**c**, *white arrow*) and persistent hyaloid vessel (*arrowhead*) connecting to the fibroplasia (*white arrow*) on the posterior surface of lens (**d**)

We then examined the fundus of the mutant mice by indirect ophthalmoscopy. Normal fundus was observed in all of the ten 2-month old wild-type mice (Fig. 2a). While out of thirteen 2-month old *Large* ^*PB/PB*^ mice, one mutant mouse had retinal vessel tortuosity (Fig. 2b), and twelve mutant mice exhibited vitreal fibroplasia (Fig. 2c). Persistent hyaloid vessels were also observed to connect with the vitreal fibroplasia in these twelve mutant mice (Fig. 2d). These features were observed as early as 1 month of age, the earliest time point of investigation, and remained unchanged in *Large* ^*PB/PB*^ mice as old as 1 year, indicating that the clinical defects of *Large* mutants are stable.

**Fig. 3:**
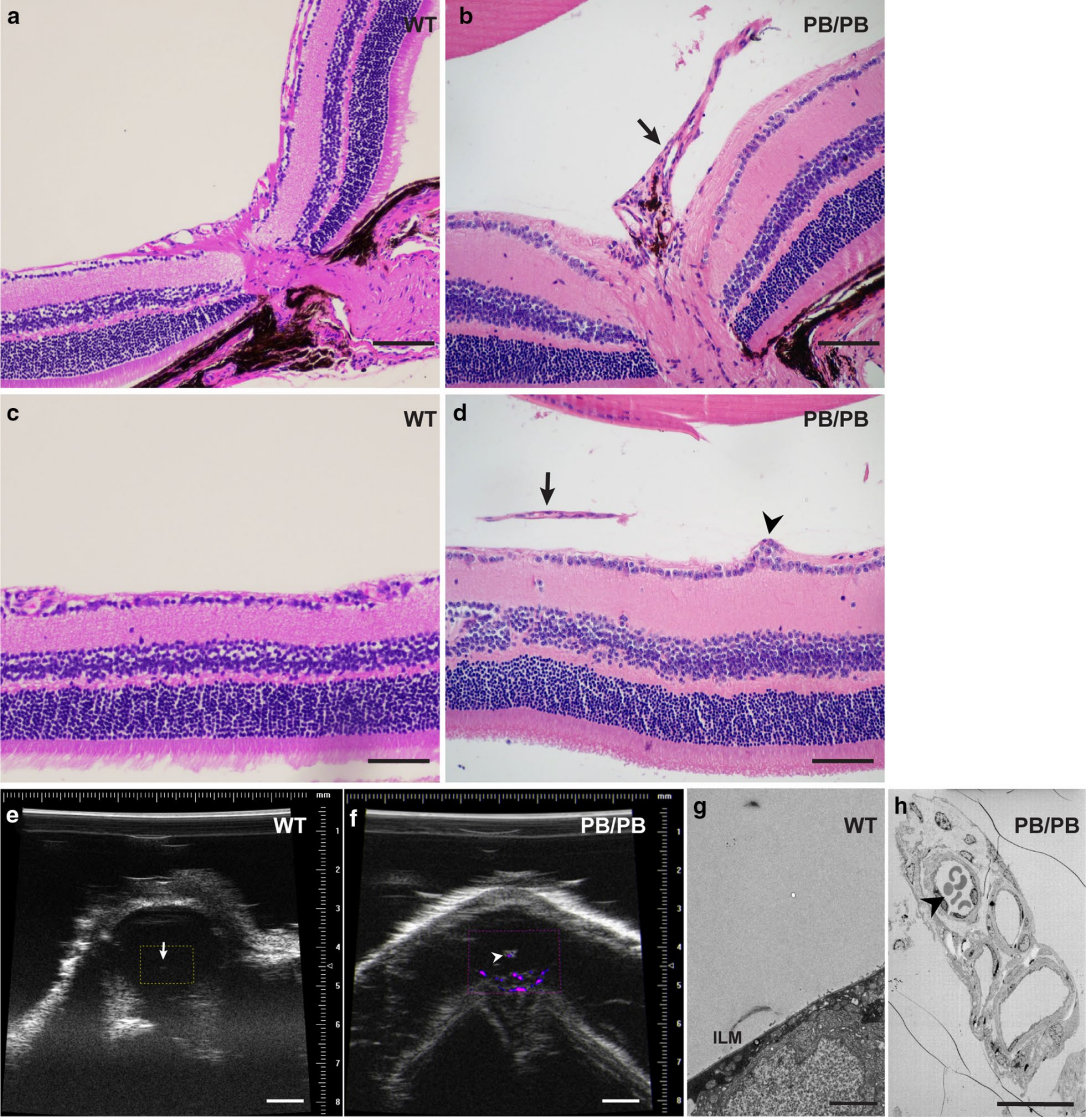
Persistent fatal vasculature (PFV) in *Large* ^*PB/PB*^ mutants. **a**–**d** Histological analysis of 2-month old wild-type and *Large* ^*PB/PB*^ mutants revealed remnant of hyaloid vessels (**b**, **d**, *arrow*) and ectopic cells anterior to inner limiting membrane (**d**, *arrowhead*) in the mutant mice. **e** Power Doppler mode of ultrasonic imaging for the eyes of 2-month old wild-type mice. No blood flow was observed around the posterior surface of the lens (*arrow*). **f** Power Doppler mode of ultrasonic imaging detected blood flow (*arrowhead*) in the persistent hyaloid vessels of 2-month old *Large* ^*PB/PB*^ mice. **g** Electron microscopy did not show any blood vessel in the vitreous of 2-month old wild-type mice. **h** Hematocytes (*arrowhead*) were observed in the persistent hyaloid vessels of 2-month old *Large* mutants under electron microscopy. *Scale bars* (**a**–**d**), 100 μm; *scale bar* (**e**, **f**), 1 mm; *scale bar* (**g**), 2 μm; *scale bar* (**h**), 20 μm

The cobweb-like vitreal fibroplasia is reminiscent of persistent fetal vasculature (PFV) in human. Further histological analysis revealed a remnant of hyaloid artery extending from the optic disc towards the posterior lens surface in 2-month old *Large* ^*PB/PB*^ mice (Fig. 3a, b), as well as ectopic cells and blood vessels in the vitreous (Fig. 3c, d). To check whether blood still flows in the remaining hyaloid vessels, we performed axial ultrasonic imaging with Power Doppler mode on 2-month old *Large* ^*PB/PB*^ mice and found a fibrovascular tissue attached to the posterior surface of the lens with blood flow signal (Fig. 3e, f). Ultrastructural examination by electron microscopy also confirmed blood cells in the ectopic vessels in the vitreous of 2-month old *Large* ^*PB/PB*^ mice (Fig. 3g, h).

### Abnormal astrocyte migration in *Large* mutant retinas

**Fig. 4:**
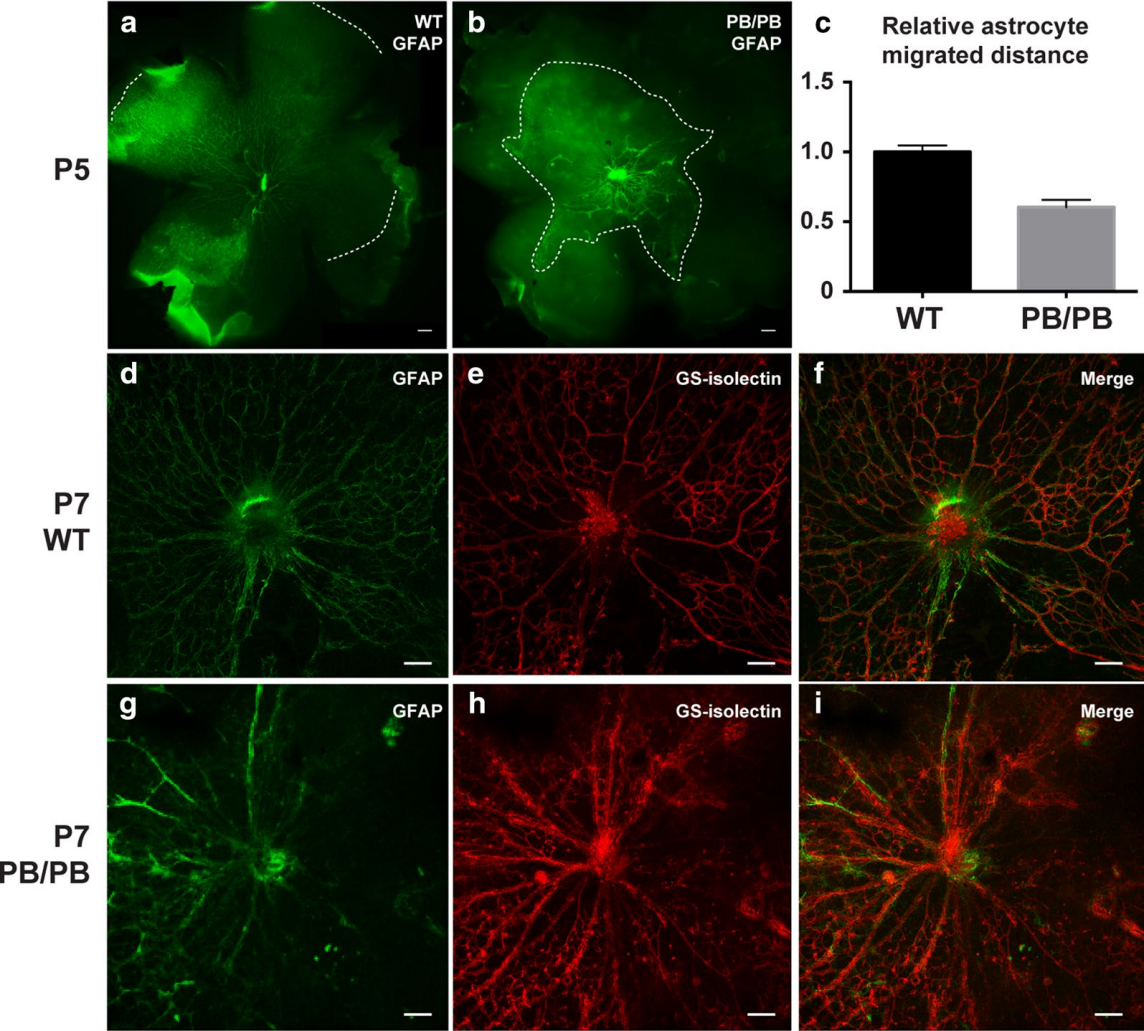
Abnormal astrocyte migration and retinal vasculature in *Large* ^*PB/PB*^ mutants. **a**–**b** GFAP staining showed normal honeycomb pattern of retinal astrocytes in P5 wild-type mice (**a**), but disorganized astrocyte processes in P5 *Large* ^*PB/PB*^ mice (**b**). *Dashed lines* indicate the border of astrocyte migration. **c** Retinal astrocytes in P5 *Large* ^*PB/PB*^ mice migrated more slowly than wild-type mice of the same age. **d**–**f** GFAP and GS-isolectin staining of P7 wild-type retinas. **g**–**i** GFAP and GS-isolectin staining of P7 *Large* ^*PB/PB*^ retinas. *Scale bars* (**a**, **b**), 200 μm; *scale bar* (**d**–**i**), 100 μm

The vessel tortuosity from indirect ophthalmoscopy also indicates abnormal retinal vasculature in *Large* ^*PB/PB*^ mice. We then checked the retinal vasculature development in *Large* ^*PB/PB*^ mice. GFAP-positive astrocytes are known to guide endothelial cell migration and retinal vasculature development [15]. On postnatal day 5 (P5), whole-mount GFAP staining showed that wild-type retinal astrocytes with a honeycomb pattern already migrated close to the peripheral region (Fig. 4a). While in P5 *Large* ^*PB/PB*^ mice, GFAP staining of astrocytes was highly irregular with large areas loss of positive staining. Astrocytic processes were disorganized and could not form the same pattern as in wild type mice (Fig. 4b). The average retinal astrocyte migration distance in *Large* ^*PB/PB*^ mice was decreased by about 40%, compared with the wild-type mice (Fig. 4c). In P7 wild-type mice, a honeycomb pattern of GFAP-positive astrocytic template was fully formed across the retina (Fig. 4d). *Griffonia simplicifolia* isolectin (GS-isolectin) highlighted the retinal blood vessels in close association with the retinal astrocytic template (Fig. 4e, f). In P7 *Large* ^*PB/PB*^ mice, although GFAP positive signal can be detected in the peripheral region, the astrocytic network was largely disturbed, leading to the abnormal retinal vasculature (Fig. 4g–i).

### Broken inner limiting membrane (ILM) in *Large* mutants

**Fig. 5:**
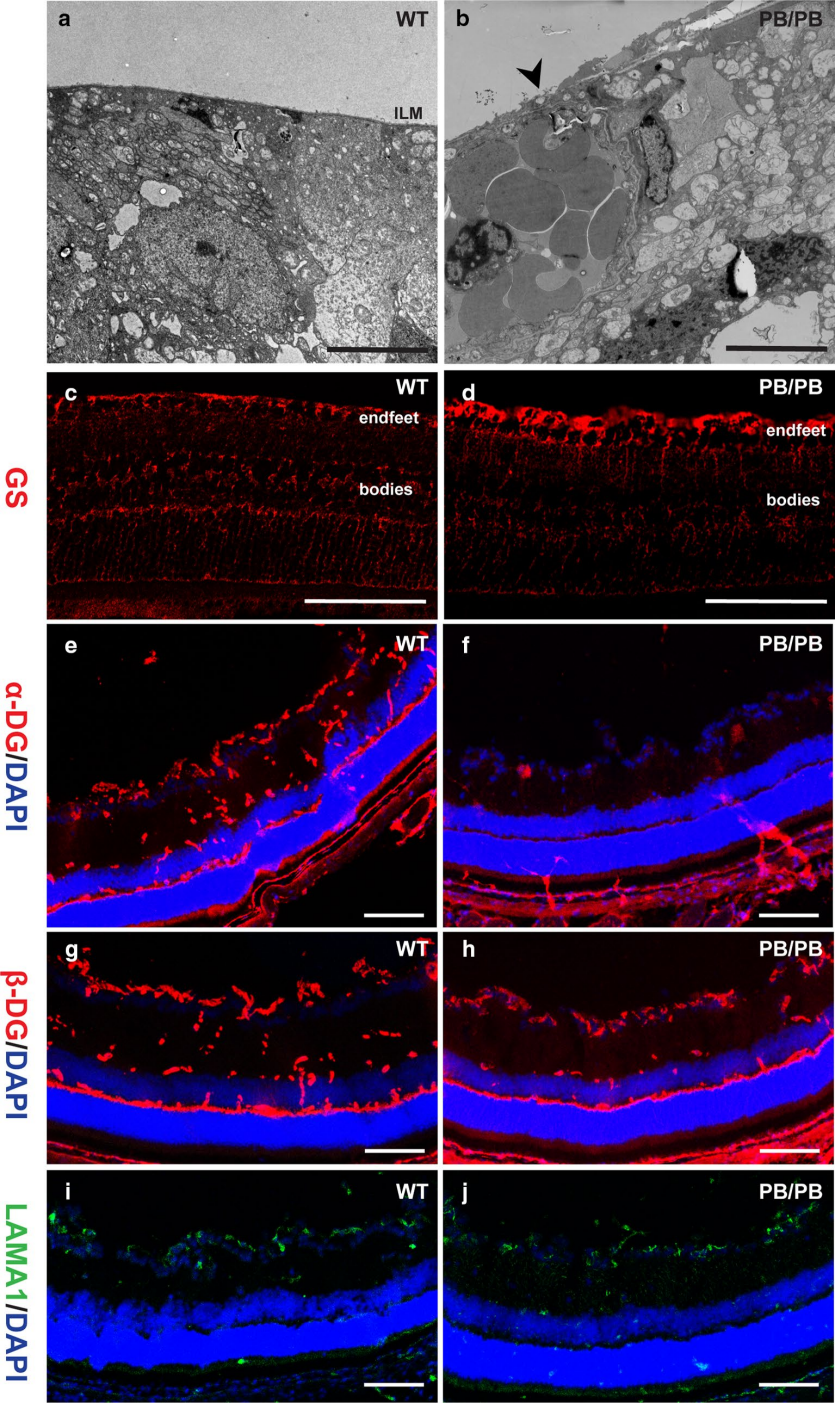
Broken ILM in *Large* ^*PB/PB*^ mutants. Compared with the intact ILM in 2-month old wild-type mice (**a**), electron microscopy examination revealed broken ILM in 2-month old *Large* ^*PB/PB*^ mice (**b**). Immunofluorescence staining for anti-glutamine synthetase (GS) showed that Müller cell end-feet reached ILM in both P22 wild-type (**c**) and *Large* ^*PB/PB*^ mice (**d**). Compared with the 2-month old control mice (**e**, **g**), glycosylated α-DG could rarely be detected in *Large* ^*PB/PB*^ retinas (**f**), but β-DG distribution seemed unaffected (**h**). **i**, **j** Laminin α1 distribution in the ILM was not affected in 2-month old *Large* ^*PB/PB*^ mice (**j**). *Scale bars* (**a**, **b**), 5 μm; *scale bars* (**c**–**j**) 100 μm

We then checked the ILM in *Large* ^*PB/PB*^ mice. H&E staining of the retinas showed that ILM was formed in *Large* ^*PB/PB*^ mice (Fig. 3b, d). However, closer examination with electron microscopy revealed focal disruptions in the ILM of 2-month old *Large* ^*PB/PB*^ mice (Fig. 5a, b), indicating that the ILM integrity was disrupted.

To determine if the Müller cell end-feet, which normally attach to ILM, were affected in *Large* ^*PB/PB*^ mice, retinal sections were stained with an antibody against glutamine synthetase (GS). On P22, Müller cell processes extended through the entire length of the retina and terminated with end-feet below the ILM in *Large* ^*PB/PB*^ mice, similar with those of age-matched wild-type mice (Fig. 5c, d).

**Fig. 6:**
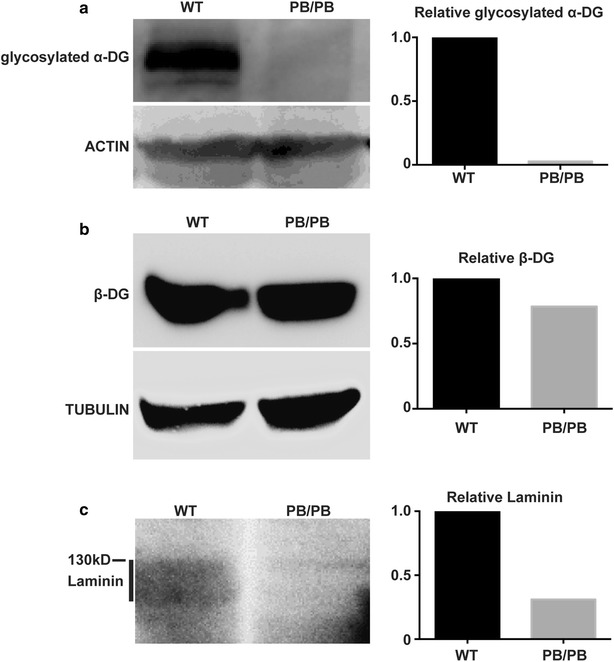
Hypo-glycosylation of α-DG in *Large* ^*PB/PB*^ mice. **a**, **b** Protein levels of α-DG (**a**) and β-DG (**b**) in 2-month old wild-type and *Large* ^*PB/PB*^ retinas. Quantification results of the bands are shown on the *right*. The α-DG antibody only recognizes glycosylated protein. **c** Laminin overlay assay showed impaired interaction between α-DG and laminin in *Large* mutant retinas

Proper glycosylation of dystroglycan is essential for the assembly and maintenance of basement membrane and epithelial structures [5]. To determine the molecular basis for the ILM defect in *Large* ^*PB/PB*^ mice, we checked the expression of two dystroglycan proteins, α-DG and β-DG, which are normally localized in outer plexiform layer (OPL) and ILM of wild-type retinas (Fig. 5e, g). Different from *Large* ^*vls*^ mice, glycosylated α-DG, which was recognized by glycosylation specific antibody (clone IIH6C4), was absent in both OPL and ILM in 2-month old *Large* ^*PB/PB*^ mice (Fig. 5e, f), while β-DG seemed unaffected (Fig. 5g, h). We also performed western blot to quantify the protein level of α-DG and β-DG in the retinas. The results confirmed the absence of glycosylated α-DG in the *Large* ^*PB/PB*^ retinas (Fig. 6a). Robust β-DG was still detected in *Large* ^*PB/PB*^ retinas (Fig. 6b). These results are consistent with the previous report that α-DG serves as a substrate of LARGE-mediated glycosylation [8].

Laminin is an essential extracellular matrix component that binds with glycosylated α-DG to guide astrocyte migration and maintain the ILM integrity [16]. We checked the expression pattern of Laminin α1 in the ILM, and did not find obvious differences between 2-month old wild-type and *Large* ^*PB/PB*^ mice (Fig. 5i, j). Since glycosylation of α-DG in *Large* ^*PB/PB*^ mice was disrupted, we assumed that the interaction between α-DG and Laminin was impaired. To prove this, we transferred 2-month old wild-type and *Large* ^*PB/PB*^ retinal protein lysate from gel to the PVDF membrane, and incubated the membrane with Laminin-1 solution. We then detected Laminin protein that bound to the membrane by western blot after extensive washing. The laminin overlay assay showed much weaker signal around the band size of α-DG in *Large* ^*PB/PB*^ group (Fig. 6c), confirming that the binding between Laminin and α-DG was impaired in *Large* ^*PB/PB*^ mice. These results indicate that the extracellular matrix is not well assembled in *Large* ^*PB/PB*^ mice, and the broken ILM in *Large* ^*PB/PB*^ mice is probably due to hypoglycosylated α-DG.

### Abnormal astrocyte migration to vitreous in *Large* mutants

**Fig. 7:**
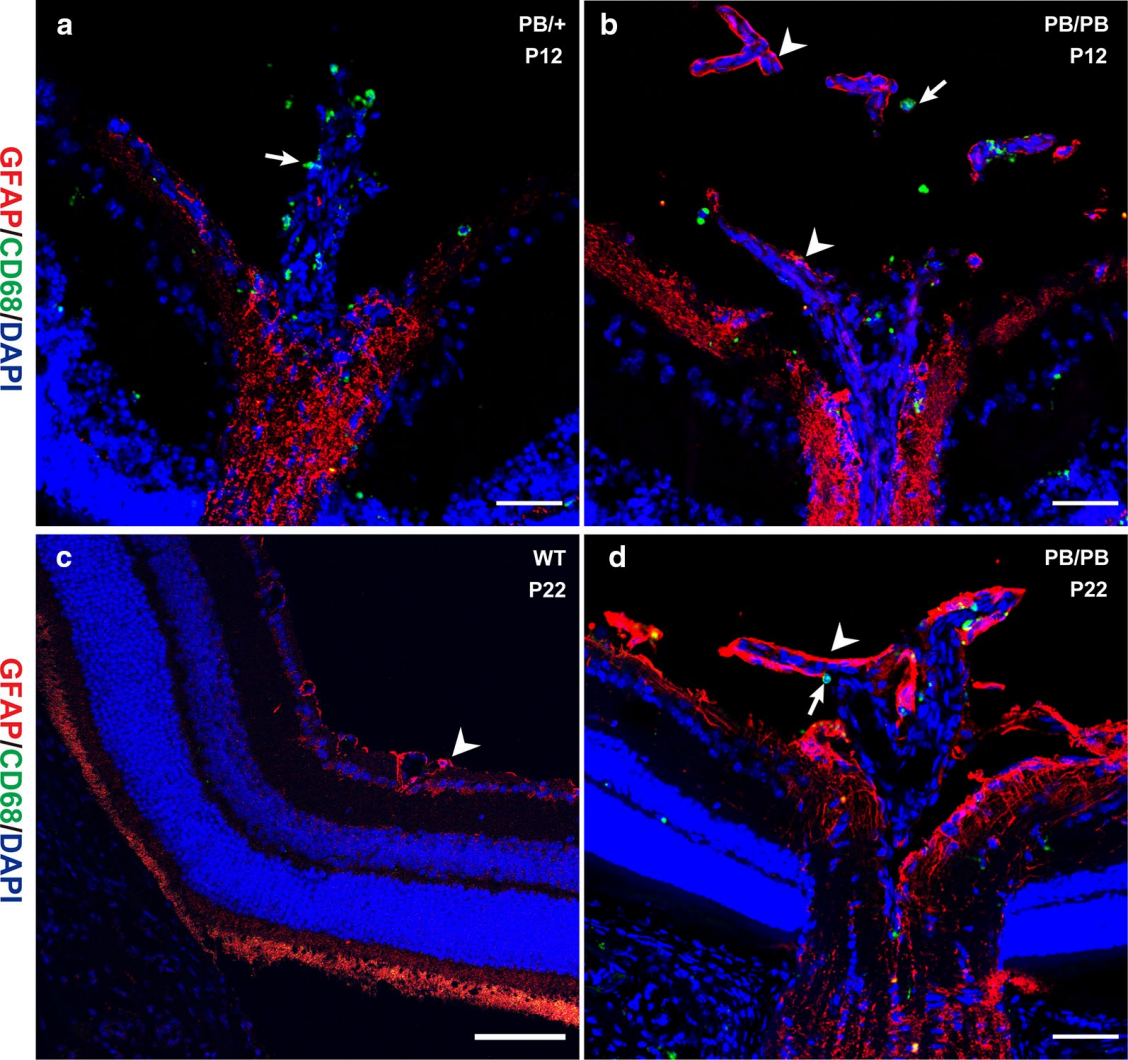
Astrocytes were associated with persistent hyaloid vessels in *Large* ^*PB/PB*^ mutants. **a** In P12 control mice, CD68-positive macrophages (*arrow*, *green signal*) were found around the regressing hyaloid vessels, and GFAP-positive astrocytes were absent in the vitreous. **b** In P12 *Large* ^*PB/PB*^ mice, except for macrophages (*arrow*, *green signal*), astrocytes (*arrowhead*, *red signal*) also migrated into the vitreous ensheathing the hyaloid vessels. **c** In P22 wild-type mice, hyaloid vessels have regressed completely. NO signal can be detected in the vitreous. *Arrowhead* indicates astrocytes in ILM. **d** Association between astrocytes (*arrowhead*) and persistent hyaloid vessels still existed in P22 *Large* ^*PB/PB*^ mice. *Arrow* indicates macrophages in the vitreous. *Scale bars*, 50 μm

Macrophages are involved in the normal regression of the hyaloid vasculature [17]. In P12 control mice, CD68-positive macrophages closely surrounded the stump of the regressing hyaloid artery that was devoid of astrocytes (Fig. 7a). While in *Large* ^*PB/PB*^ mice of the same age, the hyaloid artery was closely associated with astrocytes, with numerous macrophages in the vitreous (Fig. 7b). In P22 wild-type mice, the hyaloid artery has completely regressed (Fig. 7c). However, the association between astrocytes and remaining hyaloid vessels remained in the vitreous of P22 *Large* ^*PB/PB*^ mice (Fig. 7d). Association of astrocytes and persistent hyaloid vessels was also reported in both human PFV patients and some mouse models [3, 18, 19]. We hypothesize that the ectopic astrocytes may stabilize the vessels and inhibit normal cellular interactions that are required for programmed hyaloid regression.

## Discussion

*Large* was reported as a causative gene for human muscle–eye–brain diseases characterized by severe congenital muscular dystrophy, eye abnormalities and neuronal migration defects in central nervous system [10, 11, 20]. However, its role in retinal vasculature development remains to be explored. In this study, we reported a novel *Large* mutant that exhibited PFV and retinal vasculature defects, which is likely due to the disorganization of ILM and consequent astrocyte migration defects.

*Large* ^*myd*^ and *Large* ^*vls*^ are two previously reported mouse mutants that have exon 5–7 and exon 3–5 of *Large* deleted, respectively [7]. *Large* ^*myd*^ likely produces a truncated protein with the N-terminal transmembrane domain (TM) and coiled coil domain (CC), while *Large* ^*vls*^ likely generates a shorter truncated protein with only TM domain. RT-PCR result indicates that our *PB* allele produces the longest truncated protein with not only TM and CC, but also part of the catalytic domain. Due to the genetic differences, it’s reasonable that the ocular phenotypes in these three mutants are not exactly the same. To our knowledge, this is the first time that blood flow was observed in the persistent hyaloid vessels in the vitreous of *Large* ^*PB/PB*^ mice, while only fibroplasia was observed in *Large* ^*myd/myd*^ and *Large* ^*vls/vls*^ mutants. Besides, both α-DG and β-DG are disrupted in ILM of *Large* ^*vls/vls*^ [7], while only α-DG is affected in the *Large* ^*PB/PB*^ mutants.

In the retina, interaction between α-DG and laminins are crucial for ILM formation [16]. Mutations in Lama1, α-DG, and POMGnT1 (another enzyme involved in glycosylation of α-DG) caused abnormal laminin deposition, resulting in defective formation, abnormal astrocyte distribution, and defective blood vessel formation [3, 6]. These results support our hypothesis that the broken ILM in *Large* mutants is probably due to the impaired interaction between hypo-glycosylated α-DG and laminins.

To our knowledge, this is the first report that retinal astrocyte can migrate into the vitreous and ensheathe the persistent hyaloid vessels in *Large* mutant mice. The protective mechanism of astrocytes in the maintenance of vitreous blood vessels could be a new direction for study of the persistent hyaloid vessel. Macrophages play critical roles in programmed hyaloid vessel regression. The macrophage WNT7b serves as a short-range paracrine signal to initiate the programmed cell death in the adjacent vascular endothelial cells of the temporary hyaloid vessels of the developing eye [21]. In *Large* ^*PB/PB*^ mice, the astrocytes that ensheathe the hyaloid artery may prevent contact between the macrophages and vascular endothelial cells, thereby protecting the vascular endothelial cells from apoptosis and blocking involution of the hyaloid artery.

## Conclusions

Our results indicate that *Large* is essential for ILM formation and retinal astrocyte migration. The novel *Large* mutant mouse line can be used as a new PFV model to further dissect LARGE functions in ocular vasculature development.

## Methods

### Mouse strains

All animal experiments were performed in accordance with protocols approved by the Animal Care and Use Committee of the Institute of Developmental Biology and Molecular Medicine (IDM), Fudan University. *Large* mutant line (W146qRP) was generated by inserting a *piggyBac* transposon (PB) in *Large* during the process of a large-scale insertional mutagenesis project on the C57BL/6J background. In the *Large* ^*PB*^ allele, the PB insertion was mapped in the sixth intron (Chr: 8. 75490122, Ensembl release 54). The gene trap element in *PB* transposon contains splicing acceptor–IRES–lacZ coding sequence-polyA signal and can disrupt the expression of inserted gene efficiently. The PB insertion direction and inserted genomic sequence are also labeled in Fig. 1a.

### PCR

Genotyping PCR was performed with a *PB* specific primer LB2 (5′-CTGAGATGTCCTAAATGCACAGCG**-**3′) and two flanking genomic primers W146qRP-L1 (5′-TTCACTGCCTTTTCCTCCAGC-3′) and W146qRP-R1 (5′-CCCCACAACTTTCCTGTTCATTAC-3′). RT-PCR was performed with the following primers: Large-F 5′-ACCAAAACTCTGCCTGCCAAC-3′, Large-R 5′-CTGCTCCCATTTCATCTTCCG-3′, Gapdh-F 5′-TGTTCCTACCCCCAATGTGTCC-3′, Gapdh-R 5′-GGAGTTGCTGTTGAAGTCGCAG-3′.

### Clinical assessment

Mice were phenotyped by indirect ophthalmoscopy according to previously described methods [7].

### Histology

Retinas were dissected and embedded with OCT according to the standard protocol [3]. Hematoxylin-eosin (H&E) staining was then performed on 7-μm sections as previously described [3].

### Immunodetection assessment

Immunofluorescence staining of both cryosections and whole mount retina was performed as previously reported [7]. Primary antibodies used on cryosections included anti-alpha DG (1:200, Millipore, Cat. 05-593), anti-beta DG (1:100, Abcam, ab49515), anti-LAMA1 (1:200, Millipore, MAB1903), anti-GFAP (1:500, DAKO Z0334), and anti-CD68 (1:100, Abcam, ab31630). Primary antibodies used for whole mount staining included anti-GFAP (1:100), and anti-*G. simplicifolia* isolectin (1:200, Invitrogen).

### Imaging

Light microscopy images were collected with Leica MZFLIII or DMRXA2. Electron microscopy imaging was performed as previously described [7]. A Visualsonics Vevo 770 was used for the ultrasonic analysis of retinal defects in mutant mice.

### LAMININ overlay assay

Laminin overlay assay was performed as previously reported [10]. Briefly, PVDF membranes were incubated with TBS buffer containing 3% BSA, 1 mM CaCl_2_, and 1 mM MgCl_2_ for 1 h to block nonspecific binding. The membranes were then incubated with 1.25 µg/ml laminin-1 in TBST containing 1 mM CaCl_2_ and 1 mM MgCl_2_ overnight at 4 °C. After extensive washing, bound laminin was detected by standard Western blot procedures.

## Abbreviations

PFV: persistent fetal vasculature
*PB*: *piggyBac* transposon
ILM: inner limiting membrane
DG: dystroglycan
GS: glutamine synthetase
GS-isolectin: *Griffonia simplicifolia* isolectin
OPL: outer plexiform layer
TM: transmembrane domain
CC: coiled coil domain

## References

[1] Fruttiger M (2007). Development of the retinal vasculature. Angiogenesis.

[2] Halfter W, Dong S, Dong A, Eller AW, Nischt R (2008). Origin and turnover of ECM proteins from the inner limiting membrane and vitreous body. Eye.

[3] Edwards MM, Mammadova-Bach E, Alpy F, Klein A, Hicks WL, Roux M, Simon-Assmann P, Smith RS, Orend G, Wu J (2010). Mutations in Lama1 disrupt retinal vascular development and inner limiting membrane formation. J Biol Chem.

[4] Edwards MM, McLeod DS, Grebe R, Heng C, Lefebvre O, Lutty GA (2011). Lama1 mutations lead to vitreoretinal blood vessel formation, persistence of fetal vasculature, and epiretinal membrane formation in mice. BMC Dev Biol.

[5] Durbeej M, Henry MD, Campbell KP (1998). Dystroglycan in development and disease. Curr Opin Cell Biol.

[6] Takahashi H, Kanesaki H, Igarashi T, Kameya S, Yamaki K, Mizota A, Kudo A, Miyagoe-Suzuki Y, Takeda S, Takahashi H (2011). Reactive gliosis of astrocytes and Muller glial cells in retina of POMGnT1-deficient mice. Mol Cell Neurosci.

[7] Lee Y, Kameya S, Cox GA, Hsu J, Hicks W, Maddatu TP, Smith RS, Naggert JK, Peachey NS, Nishina PM (2005). Ocular abnormalities in Large(myd) and Large(vls) mice, spontaneous models for muscle, eye, and brain diseases. Mol Cell Neurosci.

[8] Inamori K, Yoshida-Moriguchi T, Hara Y, Anderson ME, Yu L, Campbell KP (2012). Dystroglycan function requires xylosyl- and glucuronyltransferase activities of LARGE. Science.

[9] Longman C, Brockington M, Torelli S, Jimenez-Mallebrera C, Kennedy C, Khalil N, Feng L, Saran RK, Voit T, Merlini L (2003). Mutations in the human LARGE gene cause MDC1D, a novel form of congenital muscular dystrophy with severe mental retardation and abnormal glycosylation of alpha-dystroglycan. Hum Mol Genet.

[10] Holzfeind PJ, Grewal PK, Reitsamer HA, Kechvar J, Lassmann H, Hoeger H, Hewitt JE, Bittner RE (2002). Skeletal, cardiac and tongue muscle pathology, defective retinal transmission, and neuronal migration defects in the Large(myd) mouse defines a natural model for glycosylation-deficient muscle–eye–brain disorders. Hum Mol Genet.

[11] Michele DE, Barresi R, Kanagawa M, Saito F, Cohn RD, Satz JS, Dollar J, Nishino I, Kelley RI, Somer H (2002). Post-translational disruption of dystroglycan-ligand interactions in congenital muscular dystrophies. Nature.

[12] van Reeuwijk J, Grewal PK, Salih MA, de Bernabe DBV, McLaughlan JM, Michielse CB, Herrmann R, Hewitt JE, Steinbrecher A, Seidahmed MZ (2007). Intragenic deletion in the LARGE gene causes Walker–Warburg syndrome. Hum Genet.

[13] Ding S, Wu X, Li G, Han M, Zhuang Y, Xu T (2005). Efficient transposition of the piggyBac (PB) transposon in mammalian cells and mice. Cell.

[14] Sun LV, Jin K, Liu Y, Yang W, Xie X, Ye L, Wang L, Zhu L, Ding S, Su Y (2008). PBmice: an integrated database system of piggyBac (PB) insertional mutations and their characterizations in mice. Nucleic Acids Res.

[15] Dorrell MI, Aguilar E, Friedlander M (2002). Retinal vascular development is mediated by endothelial filopodia, a preexisting astrocytic template and specific R-cadherin adhesion. Invest Ophthalmol Vis Sci.

[16] Gnanaguru G, Bachay G, Biswas S, Pinzon-Duarte G, Hunter DD, Brunken WJ (2013). Laminins containing the beta2 and gamma3 chains regulate astrocyte migration and angiogenesis in the retina. Development.

[17] Hose S, Zigler JS, Sinha D (2005). A novel rat model to study the functions of macrophages during normal development and pathophysiology of the eye. Immunol Lett.

[18] Zhang C, Asnaghi L, Gongora C, Patek B, Hose S, Ma B, Fard MA, Brako L, Singh K, Goldberg MF (2011). A developmental defect in astrocytes inhibits programmed regression of the hyaloid vasculature in the mammalian eye. Eur J Cell Biol.

[19] Hurskainen M, Eklund L, Hagg PO, Fruttiger M, Sormunen R, Ilves M, Pihlajaniemi T (2005). Abnormal maturation of the retinal vasculature in type XVIII collagen/endostatin deficient mice and changes in retinal glial cells due to lack of collagen types XV and XVIII. FASEB J.

[20] Mercuri E, Messina S, Bruno C, Mora M, Pegoraro E, Comi GP, D’Amico A, Aiello C, Biancheri R, Berardinelli A (2009). Congenital muscular dystrophies with defective glycosylation of dystroglycan: a population study. Neurology.

[21] Lobov IB, Rao S, Carroll TJ, Vallance JE, Ito M, Ondr JK, Kurup S, Glass DA, Patel MS, Shu W (2005). WNT7b mediates macrophage-induced programmed cell death in patterning of the vasculature. Nature.

